# Epidermal Growth Factor and Intestinal Barrier Function

**DOI:** 10.1155/2016/1927348

**Published:** 2016-07-25

**Authors:** Xiaopeng Tang, Hu Liu, Shufen Yang, Zuohua Li, Jinfeng Zhong, Rejun Fang

**Affiliations:** ^1^College of Animal Science and Technology, Hunan Agricultural University, Changsha 410128, China; ^2^Hunan Co-Innovation Center of Animal Production Safety, Changsha 410128, China; ^3^Hunan Polytechnic of Environment and Biology, Hengyang 421005, China

## Abstract

Epidermal growth factor (EGF) is a 53-amino acid peptide that plays an important role in regulating cell growth, survival, migration, apoptosis, proliferation, and differentiation. In addition, EGF has been established to be an effective intestinal regulator helping to protect intestinal barrier integrity, which was essential for the absorption of nutrients and health in humans and animals. Several researches have demonstrated that EGF via binding to the EGF receptor and subsequent activation of Ras/MAPK, PI3K/AKT, PLC-*γ*/PKC, and STATS signal pathways regulates intestinal barrier function. In this review, the relationship between epidermal growth factor and intestinal development and intestinal barrier is described, to provide a better understanding of the effects of EGF on intestine development and health.

## 1. Introduction

In addition to serving as a major organ for nutrient digestion and absorption, the single layer of intestinal epithelium lining the gut acts as a selective barrier to prevent the passing of toxins, allergens, and pathogens from the luminal content into the circulation system and other tissues [1]. Dysfunction of the intestinal barrier is associated with increased gut permeability and development of multiple gastrointestinal diseases, such as food allergy, inflammatory bowel disease (IBD), irritable bowel syndrome (IBS), celiac disease, and infectious enterocolitis [2–4]. Recently, several substances, such as glutamine [2], sodium butyrate [5], bile acid [6], lactic acid bacteria [7], and epidermal growth factor (EGF) [8, 9], have been reported to have a protective effect on intestinal epithelial through various underlying mechanisms.

EGF was first discovered by Dr. Cohen more than half a century ago [10]. It is a cytoprotective peptide consisting of 53 amino acid residues and three intramolecular disulfide bridges which has been detected in a variety of body fluids, such as saliva, milk, amniotic fluid, urine, plasma, and intestinal fluid, which is produced and secreted by the submaxillary salivary glands, mammary glands, placenta, kidney, and duodenal Brunner's glands, respectively [11]. EGF plays an important role in regulating cell growth, survival, migration, apoptosis, proliferation, and differentiation [12–16]. In additional to enhancing cellular proliferation and differentiation, EGF also functions as a gastrointestinal tract (GI) mucosal protective factor, which associates with intestinal maturation and maintenance of epithelial cell homeostasis in the small intestine [17]. The biological actions of EGF are mediated via binding to the EGF receptor (EGFR), a transmembrane receptor tyrosine kinase of the ErbB family, that leads to autophosphorylation of receptor tyrosine kinase (RTK) and subsequent activation of Ras/mitogen-activated protein kinases (Ras/MAPK), phosphatidylinositol 3-kinase/AKT (PI3K/AKT), phospholipase C-*γ*/protein kinase C (PLC-*γ*/PKC), and STATS signal pathways [18], to promote intestinal development [15, 19–22], regulate tight junction protein expression [9, 23–25], reduce cell autophagy [26], inhibit apoptosis induced by oxidative stress [16], and reduce the colonization of the intestinal epithelium by enteropathogens [8, 27–30].

## 2. EGF and Intestinal Development

EGF is acid- and heat-stable and resistant to proteases digestion; it can be administered orogastrically and delivered to the brush border of the small intestine segment where EGFR is abundantly located on both the apical and basolateral aspect of villus enterocytes [31]. The binding of EGF at the enterocytes surface induces dimerization of EGFR, which results in activation of EGFR tyrosine kinase activity and RTK autophosphorylation and subsequent activation of various signal transduction pathways leading to cellular proliferation and differentiation that help in intestinal development and intestinal mucosa repair [32, 33]. EGF is one of the most abundant growth factors in the milk, more than 500 times higher than other growth factors such as amphiregulin and TGF-*α* detected in human colostrums [34], indicating an important function EGF performed in early intestinal development. Previous evidence has indicated that EGF plays a significant role in intestinal development, including increasing villous height and crypt depth, enhancing enterocyte proliferation, and stimulating secretion of digestive enzymes such as trypsin, chymotrypsin, alkaline phosphatase, sucrase, maltase, and lactase, which is important for improving nutrition absorption, feed utilization, and growth performance of animals [15, 19–22, 35–39]. The applications of EGF for animals are listed in Table 1.

The intestinal development is related to the intestinal barrier integrity directly, to keep intestinal health, and intracellular homeostasis is essential for the formation of the intestinal barrier. Pervious study has shown EGF controlling mucosal homeostasis through regulating the tight junction components [9, 40], enhancing the mucins secretion [41, 42], and decreasing pathogens colonization [8, 27–30].

## 3. EGF and Intestinal Barrier

### 3.1. Intestinal Barrier Structure

The intestinal epithelium is formed by a continuous monolayer of proliferating and differentiating intestinal epithelial cells (IECs) separating the intestinal mucosa from the lumen environment. The IECs are tightly bound together by junctional complexes (including tight junctions, gap junctions, adherens junctions, and desmosomes [43, 44]), which form a selective barrier that allows nutrients absorption and defends against toxins, allergens, and pathogens from the gut lumen into mucosal tissue and circulation [45]. Tight junctions (TJs) seal the space between adjacent epithelial cells near the apical surface, which are the most apical components of intercellular junctional complexes [44, 46]. Adherens junctions (AJs) are located beneath the TJs and are involved in cell-cell adhesion, intracellular signaling, and the integrity of TJs regulation [24, 44, 46, 47]. Gap junctions and desmosomes contribute to cell-cell adhesion and intracellular communication, respectively [44, 46]. Disruption of the intercellular junctional complex has been reported to increase intestinal permeability that results in an easy passing of pathogens into intestinal mucosa which causes numerous gastrointestinal diseases [44, 48]. Thus, maintaining the integrity of intercellular junctional complex is critical for intestinal development and health.

### 3.2. Regulation of Tight Junction by EGF

**Figure 1:**
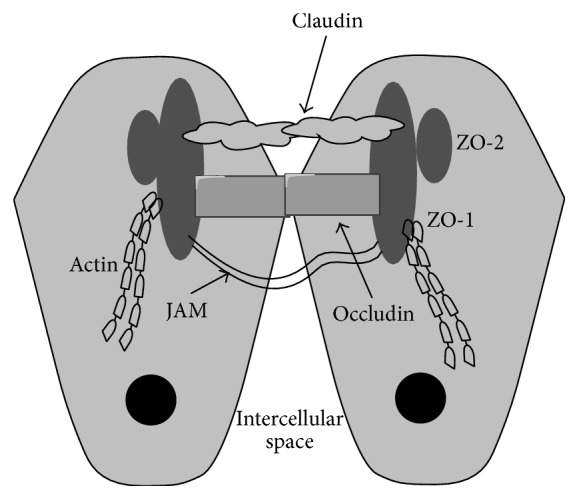
Tight junction structures. The tight junction is organized by multiple transmembrane proteins, including junctional adhesion molecules (JAM), occludin, claudins, and zona occludens (ZO), which interact in a coordinated manner to form intestinal barriers.

TJs are multiple protein complexes composed of at least three types of transmembrane proteins, claudins, occludin, and junctional adhesion molecule (JAM), which interact with cytoplasmic scaffold protein such as zona occludens (ZO-1, ZO-2, and ZO-3) and interact in a coordinated manner to form intestinal barriers (Figure 1) [61, 62]. They regulate the paracellular passage of ions, water, and solutes and act as a fence to maintain cell polarity by blocking the free diffusion of proteins and lipids between the apical and basolateral domains of the plasma membrane [62]. Significant evidences indicate that TJs are associated with numerous intracellular signaling molecules regulated by the activity of signal transduction pathways [23]. The integrity of the TJ is regulated by PKC, PI3K, MAPK, myosin light chain kinase (MLCK), the Rho family of small GTPases, G-proteins, c-Src, PLC-*γ*, and protein phosphatase 2A (PP2A) [23, 46, 63].

EGF is a key regulator of epithelial paracellular permeability, a property that depends on TJs and can be evaluated through the measurement of the transepithelial electrical resistance (TER) [23–25]. EGF has been shown to protect intestinal barrier function by preventing early-weaned [22], hydrogen peroxide [40, 50–52], ethanol [8], acetaldehyde [53–55], and intestinal ischemia-reperfusion [16, 64] induced disruption of TJs and paracellular permeability. EGF induces changes in the composition of TJ through activating several signaling pathways such as PKC [50], MAPK [23], and STATs [25] in different types of cells (Table 2).

**Figure 2:**
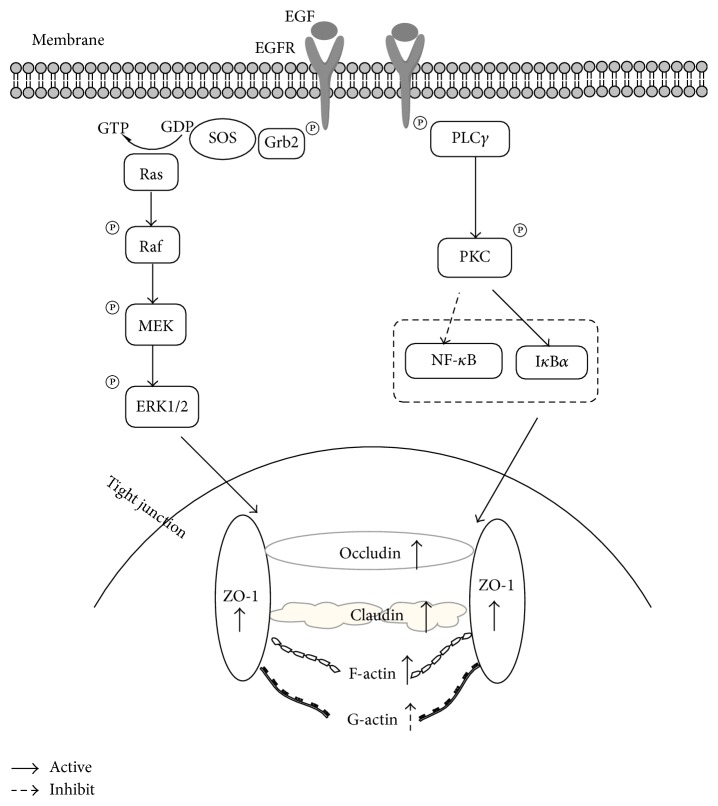
The EGFR-phospholipase (PLC)-*γ*-PKC and EGFR-ERK/MAPK signaling pathways are involved in EGF-mediated protection of tight junctions. ERK/MAPK pathways were mainly involved in regulating barrier function by improving the gene expression of tight junction proteins such as ZO-1, claudin-1, and occludin, while PLC-*γ*-PKC pathways were mainly involved in regulating actin cytoskeletal architecture such as F-actin and G-actin.

Numerous researches have demonstrated that oxidative stress impairs intestinal barrier function [65]. Weaning pigs from the sows is one of the most stressful events in the pig's life that can contribute to intestinal dysfunctions [66]. Xu et al. [22] indicated that the oral administration of EGF could improve the gene expression of tight junction proteins such as ZO-1, claudin-1, and occludin, thus enhancing the intestinal barrier function of early-weaned piglets. EGF prevented hydrogen peroxide-induced intestinal barrier disruption through ERK/MAPK and PLC/PKC pathways (Figure 2). Basuroy et al. [23] showed that, in Caco-2 cells, pretreating with EGF can inhibit the oxidative stress-induced intestinal barrier disruption, as indicated by TER, and TJ proteins (ZO-1 and occludin) redistribution, while pretreatment of Caco-2 cells with MAPK/ERK kinase (MEK) inhibitors completely blocked the protective effects of EGF on TJs. When epithelial cells suffered from stress, upon supplementation with EGF they bind to EGFR, leading to autophosphorylation of RTK; the interaction between EGFR and SHC/Grb2 results in the recruitment of SOS to the plasma membrane to activate Ras. Activated Ras mediates Raf activation and then activates MEK, leading to the activation of ERKs [18, 33]. Activated ERK can regulate the expression of TJs such as ZO-1, occludin, and claudin (Figure 2). Pretreating with EGF can increase F-actin expression, decrease G-actin expression [40], and increase the F-actin-to-G-actin ratio [52]. EGF protection against oxidants requires PKC (isoforms *β*1 and *ξ*) activation [50, 51]; the activation of PLC-*γ*/PKC-*β*1 can inhibit the activation of NF-*κ*B and enhance I*κ*B*α* stabilization, which helps to protect the F-actin assembly and barrier function in enterocyte monolayers [40, 52]. Arda-Pirincci and Bolkent [16] reported that EGF treatment of rats with ischemia-reperfusion prevented the ischemia/reperfusion-induced oxidative injury by reducing apoptosis and lipid peroxidation and by increasing antioxidant enzyme activities. Geng et al. [64] showed that the TJs (ZO-1 and occludin) in jejunum and ileum are notably accelerated and expressed in all EGF-treated ischemia-reperfusion rats. Ethanol and its oxidized metabolite, acetaldehyde, also induce intestinal hyperpermeability, which contributes to the development of alcoholic liver disease (ALD) [62]. Banan et al. [67] showed that ethanol induces disruption of the F-actin cytoskeleton and of intestinal barrier integrity, in part, through I-kBa degradation and NF-kB activation. Chen et al. [8] demonstrated that EGF improved the intestinal integrity by lowering intestinal permeability under chronic ethanol exposure. However, whether EGF protects intestinal barrier function through preventing ethanol-induced disruption of TJs and paracellular permeability has not been reported yet. Acetaldehyde, a metabolic product of ethanol oxidation, seriously harms the intestinal barrier function. Previous studies have shown that acetaldehyde, but not ethanol, disrupts TJ and increases paracellular permeability by a tyrosine kinase-dependent mechanism [24, 54, 55]. Acetaldehyde induces tyrosine phosphorylation of occludin, ZO-1, E-cadherin, and *β*-catenin and dissociates these proteins from the actin-rich, detergent-insoluble fractions [24, 53–55]. EGF prevents acetaldehyde-induced increase in paracellular permeability (as indicated by increased TER and decreased macromolecule flux) and redistribution of occludin, ZO-1, E-cadherin, and *β*-catenin from the intercellular junctions through the activation of EGFR-PLC-*γ*-PKC*β*1/*ϵ* and EGFR-ERK/MAPK signaling pathways (Figure 2) [24, 55].

Previous studies indicated that EGF has a potential role in the prevention of necrotizing enterocolitis- (NEC-) induced TJs disruption in neonates, including humans and rats [68, 69]. Clark et al. [41] showed that NEC rats supplemented with EGF can make the expression of occludin and claudin-3 in the ileum normalized, which help to maintain intestinal barrier function.

### 3.3. EGF Promotes Mucin Secretion

**Figure 3:**
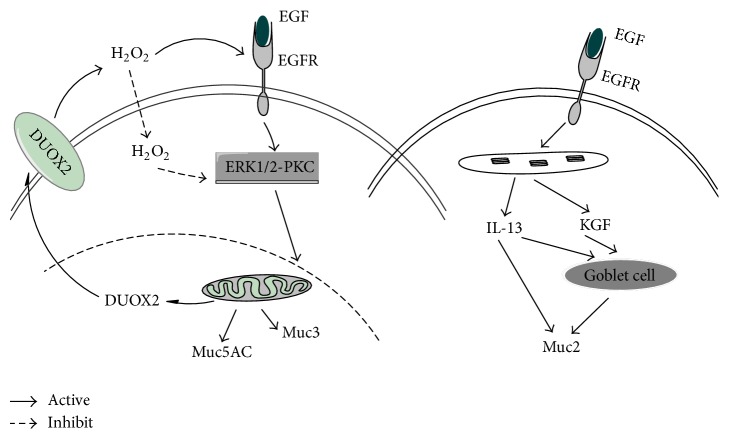
Mechanism of EGF induced mucins secretion. EGF treatment can increase the expression of interleukin-13 and keratinocyte growth factor (KGF) that resulted in an increased Muc2 expression [15]. EGF through inducing DUOX2 expression and ROS production to activate ERK1/2-PKC pathways thus inducing Muc5AC and Muc3 expression [42].

The intestinal epithelial monolayer also protects and separates itself physically from exogenous stress by secreting mucins to form a thick protective layer of mucus over the intestinal mucosas which are important for intestinal lubrication, limiting bacteria adhesion and maintaining proper intestinal permeability [15, 70]. Mucins (Muc), both secretory type (including Muc2, Muc5AC, Muc5B, Muc6, and Muc19) and membrane-bound type (including Muc1, Muc3, Muc4, Muc12, Muc13, Muc15, Muc16, Muc17, and Muc20), are high molecular weight, heavily glycosylated proteins. EGF seems to exert beneficial effects on intestinal mucosas mucin secretion especially Muc2, Muc3, and Muc5AC [15, 42]. Muc2 is one of the most predominant gel-forming mucins secreted by goblet cells in the small intestine and colon [71]. Muc5AC expressed by goblet cell is mainly present at the inner mucous layer of gastric mucosa [71]. Muc3 is a transmembrane mucin expressed in the small intestine and colon [42, 71]. Clark et al. [41] demonstrated that treatment of NEC with EGF increased goblet cell density and Muc2 production in the ileum but had no effect on Muc2 production in the jejunum. Bedford et al. [15] showed that EGF treatment can increase the expression of interleukin-13 (IL-13), stimulating both goblet cell differentiation and mucin secretion in the intestine [72], and keratinocyte growth factor (KGF), stimulating human colonic epithelial cell differentiation into goblet cells [73], that resulted in an increased Muc2 expression. The reactive oxygen species (ROS) generated by the membrane NADPH oxidase (NOXs) enzymes, such as dual oxidases 2 (DUOX2), has shown to contribute to promoting receptor signaling activation [74]. Damiano et al. [42] found that EGF modulates DUOX2 levels through ERK1/2-PKC pathways increasing ROS levels, in turn, inducing gel-forming Muc5AC and the transmembrane Muc3 expression. In conclusion, EGF through stimulating goblet cell differentiation produces Muc2 and through inducing DUOX2 expression and ROS production activates ERK1/2-PKC pathways, thus inducing Muc5AC and Muc3 expression, to form a thick protective layer of mucus over the intestinal mucosas to maintain intestinal 14 barrier integrity (Figure 3).

### 3.4. EGF Reduces Bacterial Colonization

The intestinal microbiota profile plays an essential role in intestinal integrity. EGF can reduce colonization of the intestinal epithelium by enteropathogens, such as* Escherichia coli (E. coli)* [21, 27, 59, 60],* Campylobacter jejuni (C. jejuni)* [30], and* Enterococcus* [21] (Table 3). Administration of EGF to newborn rabbits can significantly reduce bacterial translocation and was associated with increased goblet cells in intestine [59]. Oral administration of EGF to weaned rabbits infected with enteropathogenic* E. coli* showed a significant inhibition of* E. coli* colonization in the small and large intestine without affecting the proliferation of* E. coli in vitro* [27]. In addition, EGF can reduce* C. jejuni* colonization in the jejunum of* C. jejuni* infected chicks and prevent* C. jejuni*-induced claudin-4 disruption [30]. What is more, EGF showed a protective effect on TJs in experimental* Clostridium difficile (C. difficile)* infected mice [9], suggesting a potential role of EGF in reducing* C. difficile* colonization.

## 4. Conclusions

The biological functions of EGF are mediated through binding to EGFR and inducing RTK autophosphorylation and subsequent activation of various signal transduction pathways to regulate intestinal development, TJs expression, and mucins secretion which are important for the formation of intestinal barrier functions. In conclusion, EGF acts as a key epithelial mucosa regulator to regulate intestinal permeability and intestinal barrier integrity through the following 3 ways: (1) activating EGFR-PLC-*γ*-PKC and EGFR-ERK/MAPK signaling pathways to regulate TJs expression; (2) stimulating goblet cell differentiation to produce Muc2 and inducing DUOX2 expression and ROS production to activate ERK1/2-PKC pathways thus inducing Muc5AC and Muc3 expression; (3) reducing bacterial colonization and translocation.

## Figures and Tables

**Table 1:** The applications of EGF for animals on intestinal development.

Animal	Dose	Significant results	Reference
Fetal rabbit	300 *µ*g/kg/d	EGF infusion significantly increased intrauterine growth retardation, fetal small intestinal villus height, and crypt cells	[35]
Early-weaned pigs	1.5 mg/kg	Increased the mucosa IgA levels and crypt depth at jejunum on day 28 after weaning	[36]
Early-weaned mice	50 *µ*g/kg	Increased mean villous height and crypt depth and enhanced enterocyte proliferation	[37]
1-day-old, large white-duroc cross breed piglets	10** ** *µ*g/kg/d	Stimulates proliferation of intestinal crypt epithelial cells and promotes recovery from atrophic enteritis in PEDV-infected piglets	[49]
Early-weaned pigs	1.0** **mg/kg diet	Failed to alter the small intestinal villus morphology, DNA, or protein content of gastrointestinal mucosa	[19]
Early-weaned pigs	50** ** *µ*g/kg** **BW/d	Greater jejunal and duodenal villus heights; greater intestinal length	[20]
Early-weaned pigs	180** ** *µ*g/d	Increased villous height in the duodenum, jejunum, and ileum	[21]
Early-weaned pigs	115** ** *µ*g/kg** **BW/d	Enhanced jejunal structure development, increased villi height, and decreased lamina propria width	[15]
Early-weaned pigs	180** ** *µ*g/d	Increased villus height and increased the intestinal structural integrity proteins expression	[22]
Early-weaned pigs	60** ** *µ*g/kg** **BW/d	Enhanced mean villous height, crypt depth, and villous height: crypt depth and stimulated proliferation of piglet enterocytes	[38]
Early-weaned rats	50** ** *μ*g/kg	Enhanced mean villous height, crypt depth, total protein, DNA, and RNA and stimulated enterocytes proliferation	[39]

**Table 2:** The effects of EGF on EGF-mediated protection of tight junctions.

Cell lines	Inducer	TJ associated proteins	Involved pathways	Reference
Caco-2	Hydrogen peroxide	Increased in tubulin polymerization	PKC-*β*1	[50]
Caco-2	Hydrogen peroxide	Increased in tubulin polymerization and decreased in monomeric tubulin	PKC-*ξ*	[51]
Caco-2	Hydrogen peroxide	Increased F-actin-to-G-actin ratio	PKC-*β*1 ↑; NF-*κ*B ↓^2^	[52]
Caco-2	Acetaldehyde	Occludin ↑; ZO-1 ↑^1^	Inhibited tyrosine phosphorylation	[53]
Caco-2	Hydrogen peroxide	F-actin ↑; G-actin ↓	PLC-*γ* ↑; NF-*κ*B ↓	[40]
Caco-2	Hydrogen peroxide	Occludin ↑; ZO-1 ↑	ERK-MAPK	[23]
Caco-2	Acetaldehyde	Occludin ↑; ZO-1 ↑	Not mentioned	[54]
Caco-2	Acetaldehyde	Occludin ↑; ZO-1 ↑	PLC-*γ*/PKC	[55]
Caco-2	Acetaldehyde	Occludin ↑; ZO-1 ↑	ERK1/2-MAPK	[24]
NRC-1 cells^3^	Hydrogen peroxide	ZO-1 ↑; claudin-3 ↑	PLC-*γ*/PKC	[56]
MCAS^4^	None	Claudin-3 ↓	MEK/ERK or PI3K/Akt	[57]
HUOA^5^		Claudin-4 ↓		
MDCK^6^	None	Claudin-4 ↑	MEK/ERK	[58]
MDCK	None	Claudin-2 ↓; claudin-4 ↑	Src and STAT3	[25]

^1^Symbols ↑ and ↓ indicate increases and decreases in the protein or mRNA expression, respectively. ^2^Symbols ↑ and ↓ stand for activation and inhibition, respectively. ^3^Cholangiocytes. ^4^Mucinous cystadenocarcinoma. ^5^Serous cystadenocarcinoma. ^6^Darby canine kidney cells.

**Table 3:** Effects of EGF on intestinal bacterial colonization and translocation.

Animals	Significant results	Reference
New Zealand white rabbits	EGF treatment significantly inhibits enteropathogenic *Escherichia coli *colonization in the small and large intestine	[27]
Newborn rabbits	Administration of EGF significantly reduced bacterial translocation and was associated with increased goblet cells in intestine	[59]
Rats	Administration of EGF significantly reduced aerobic bacterial colonization	[60]
White leghorn chicks	EGF reduced *Campylobacter jejuni *colonization in the jejunum and dissemination to the liver and spleen and inhibited* Escherichia coli *translocation	[30]
Rats	An intervention with EGF decreased fecal *Escherichia coli* colonization	[8]
Early-weaned piglets	EGF treatment decreased the amount of* Escherichia coli* in the ileum and cecum and *Enterococcus* counts in the ileum	[21]
